# YOLOv7-TS: A Traffic Sign Detection Model Based on Sub-Pixel Convolution and Feature Fusion

**DOI:** 10.3390/s24030989

**Published:** 2024-02-03

**Authors:** Shan Zhao, Yang Yuan, Xuan Wu, Yunlei Wang, Fukai Zhang

**Affiliations:** School of Software, Henan Polytechnic University, Jiaozuo 454000, China

**Keywords:** object detection, traffic sign detection, YOLOv7, sub-pixel convolution, feature fusion

## Abstract

In recent years, significant progress has been witnessed in the field of deep learning-based object detection. As a subtask in the field of object detection, traffic sign detection has great potential for development. However, the existing object detection methods for traffic sign detection in real-world scenes are plagued by issues such as the omission of small objects and low detection accuracies. To address these issues, a traffic sign detection model named YOLOv7-Traffic Sign (YOLOv7-TS) is proposed based on sub-pixel convolution and feature fusion. Firstly, the up-sampling capability of the sub-pixel convolution integrating channel dimension is harnessed and a Feature Map Extraction Module (FMEM) is devised to mitigate the channel information loss. Furthermore, a Multi-feature Interactive Fusion Network (MIFNet) is constructed to facilitate enhanced information interaction among all feature layers, improving the feature fusion effectiveness and strengthening the perception ability of small objects. Moreover, a Deep Feature Enhancement Module (DFEM) is established to accelerate the pooling process while enriching the highest-layer feature. YOLOv7-TS is evaluated on two traffic sign datasets, namely CCTSDB2021 and TT100K. Compared with YOLOv7, YOLOv7-TS, with a smaller number of parameters, achieves a significant enhancement of 3.63% and 2.68% in the mean Average Precision (mAP) for each respective dataset, proving the effectiveness of the proposed model.

## 1. Introduction

With the rapid development of Artificial Intelligence (AI) and Deep Learning (DL) technology, traffic sign detection, as a part of intelligent transportation, has become a popular research topic. In real-world scenes, traffic sign detection can assist drivers in accurately assessing information and reduce cases of neglect and misidentification caused by fatigue or inattention to avoid accidents. However, practical traffic sign detection can be affected by several factors, such as illumination, bad weather, and occlusion. Therefore, it is important to design a robust traffic sign detector with high detection accuracy.

For increased driver perceptibility, traffic signs usually have striking colors and special shapes. Traditional traffic sign detection focuses on feature extraction and classification. Color or shape features are extracted by different approaches for traffic sign recognition through a classifier. Benallal et al. [1] performed traffic sign detection through color segmentation by comparing the differences between the RGB components. However, the RGB color space is highly sensitive to changes in the lighting environment, making it difficult to achieve stable results. Ruta et al. [2] employed a normalization process to enhance the RGB color space, to better extract and detect the differences between the three colors (red, yellow, and blue). Nguwi et al. [3] converted traffic sign images from the RGB to the HSI color space and segmented the images with a fixed threshold due to the insensitivity of the latter to illumination changes. Fleyeh et al. [4] employed an AdaBoost binary classifier-based color segmentation approach. Yang et al. [5] proposed a model based on a color probability map in the Ohta space. Loy et al. [6] attempted to establish the possible center of mass location through the symmetry of the image and the edge information to detect traffic signs. However, this method does not apply to all shapes and has poor generalization. The Hough transform approach utilized by Zaklouta et al. [7] to extract specific shape features achieved better detection results. Abukhait et al. [8] utilized both traffic sign color and shape features and demonstrated a strong detection performance, even when the traffic sign shape was distorted or obscured by foreign objects. Although traditional methods for traffic sign detection demonstrated satisfactory performance at the time, they still face the following limitations: Firstly, traditional approaches often rely on manually designed features to represent traffic signs. This may overlook some complex textures, shapes, or background variations since manually designed features struggle to capture intricate linear relationships. Secondly, factors such as lighting conditions, different perspectives, and occlusion pose challenges to traditional traffic sign detection. Under these circumstances, traffic signs exhibit varying appearances, making it difficult for traditional methods to adapt to such changes. Thirdly, due to the diversity in the appearance of traffic signs and backgrounds, traditional methods struggle to generalize across different scenes and datasets, challenging their robustness in practical applications. Lastly, the computational complexity of traditional methods is relatively high, rendering them unsuitable for meeting real-time requirements.

Since the achievements of AlexNet [9] in the field of visual recognition in 2012, an increasing number of DL methods have been proposed and applied to object detection, replacing the traditional algorithms. These DL-based object detection methods can be broadly categorized into two groups, namely Two-Stage and One-Stage algorithms. The first ever Two-Stage object detection algorithm with industrial-grade accuracy, Regions with CNN features (R-CNN), was introduced by Girshick et al. [10]. A selective search approach was utilized by R-CNN to extract 2000 candidate regions, which were subsequently predicted individually. However, despite its breakthrough accuracy, R-CNN is plagued by significant speed limitations due to the generation of candidate regions and extensively repetitive computations. The efficiency of R-CNN has been optimized by Spatial Pyramid Pooling in Deep Convolutional Networks for Visual Recognition (SPP-Net) [11], which extracts features from the entire feature map and uses a spatial pyramid pooling layer to handle features of arbitrary sizes. The SPP-Net training process remains complex, although it has been improved compared with R-CNN. Fast R-CNN [12] unifies the classification and regression problems with a deep network implementation, eliminating the need for additional storage and dramatically improving the training speed. Faster R-CNN [13] became the first object detector with near real-time performance, integrating the four main steps, i.e., feature extraction, candidate region generation, regression of predicted edge position parameters, and object category determination, into the Region Proposal Network (RPN). Libra R-CNN [14] solves the imbalance problem during training with high accuracy improvement by introducing Intersection over Union (IoU) balanced sampling, balanced pyramid, and balanced Mean Absolute Error (L1 loss). Despite the significant enhancements in detection performance brought about by these Two-Stage algorithms, their speed still does not meet the demands of real-time object detection. This issue is tackled by the introduction of One-Stage object detection algorithms. You Only Look Once (YOLO) [15] transforms object detection into a regression problem and adopts CNNs to complete the prediction of bounding boxes and category determination, truly ushering in the era of real-time object detection. A detection accuracy on par with Faster R-CNN is achieved by Single Shot MultiBox Detector (SSD) [16], while maintaining the detection speed through the prediction of multi-scale features and the introduction of an anchor mechanism. In YOLOv2 [17], batch normalization is incorporated into the network to expedite model convergence, and the K-Means algorithm is utilized to determine the size and position of prior frames, resulting in better-matching detection frames for real objects. Darknet-53 is employed as the YOLOv3 [18] backbone, and the concept of Feature Pyramid Network (FPN) [19] is introduced for feature fusion at different scales, further enhancing detection performance. YOLOv4 [20] employs a new backbone, CSPDarknet53, by combining the Cross Stage Partial Network (CSPNet) [21] with Darknet-53. This model additionally incorporates Spatial Pyramid Pooling (SPP) and Path Aggregation Network (PANet) [22] to expand the receptive field of features and improve feature fusion. In YOLOv5 [23], the feature fusion capabilities are enhanced by incorporating the CSP structure into the fusion network. Simultaneously, the Focus structure is added to the backbone network to perform slicing operations on feature maps, reducing algorithm computation and accelerating processing speed. Gui et al. [24] achieved a more lightweight model in YOLOv5 by introducing lightweight convolutional operations. Simultaneously, the incorporation of the bottleneck attention module was employed to extract effective information, thereby enhancing the accuracy of the model. Recently, breakthroughs have been achieved in the field of computer vision with models based on Transformer [25] architecture. The Vision Transformer (ViT) [26] applies the Transformer architecture to sequences of image patches, further advancing image classification tasks. ViT-FRCNN [27] integrates ViT with RPN to accomplish object detection tasks based on the transformer backbone. Xie et al. [28] proposed a novel integrated network, ViT-MVT, designed for joint optimization of image classification tasks and other downstream tasks, such as object detection and semantic segmentation. ViT-MVT demonstrates exceptional performance across multiple visual tasks, surpassing existing methods while requiring considerably less total storage. Wang et al. [29] proposed an MDL-NAS framework based on ViT, achieving high performance across various vision tasks and maintaining storage efficiency for model deployment through a coarse-to-fine searching space design and a joint-subnet search algorithm.

As a sub-type of object detection, traffic sign detection can benefit from the application of DL-based algorithms. However, this task has higher requirements for detection accuracy and speed. As a result, various studies have attempted to improve these algorithms in order to achieve improved traffic sign detection. Gavrilescu et al. [30] revealed that compared with Fast R-CNN, Faster R-CNN exhibited superior real-time traffic sign detection performance. Based on Faster R-CNN, Yang et al. [31] proposed a method utilizing an attention network to replace the RPN, improving the accuracy for both the TT100k [32] and BTSD [33] datasets. Additionally, Zhang et al. [34] introduced a cascaded R-CNN [35] approach to acquire multi-scale features from the pyramid and weighted them through dot products and softmax operations, thereby emphasizing traffic sign features and enhancing detection accuracy. The aforementioned algorithms are all formulated based on the Two-Stage object detection approach, and despite the significant improvement in accuracy, meeting real-time requirements is challenging due to the slower speed inherent in Two-Stage object detection. Mohd-Isa et al. [36] incorporated SPP into the YOLOv3 framework, enabling traffic sign recognition for real-world images of varying sizes through multi-scale pooling. This modification results in improved detection accuracy along with increased detection speed. Wang et al. [37] developed a lightweight YOLOv4 algorithm by replacing the backbone with a lightweight architecture, MobileNetv2 [38], and attention mechanisms were also employed to enhance the model’s detection capabilities. In summary, DL-based traffic sign detection algorithms are characterized by increased robustness and higher detection accuracy compared with traditional methods. Simultaneously, One-Stage algorithms offer advantages in real-time processing, better aligning with the requirements of traffic sign detection.

Despite the better traffic sign detection results and faster speeds offered by the YOLO-series-based One-Stage algorithms, the problem of the high leakage rates of small objects persists. Detecting small objects has always been a challenging task in object detection. Firstly, small objects typically exhibit low-resolution with limited visual information, making the extraction of effective features more challenging. Secondly, detection methods based on neural networks typically extract deep-level features with rich semantic information for object detection. The dimensions of these features progressively diminish, causing the network to gradually lose spatial resolution and detailed information during the forward propagation process, thereby affecting the detection of small objects. In recent years, several methods have been proposed to achieve breakthroughs in the field of small object detection. Liang et al. [39] introduced FS-SSD as a method for small object detection in UAV images. However, the algorithm introduces hyperparameters that are not conducive to optimization. Liu et al. [40] employed contextual information, encompassing object and scene features, as well as relationships between coexisting objects, to enhance the performance of small object detection. The approach relying on context fusion enhances the accuracy of object detection to a certain extent. However, identifying contextual information from a global scene that is beneficial for enhancing small object detection remains a challenging research problem. Li et al. [41] employed generative adversarial learning by mapping the features of small low-resolution objects into features equivalent to those of high-resolution objects to attain the same detection performance as that of larger-size objects. Nevertheless, training generative adversarial networks poses challenges, and achieving a well-balanced relationship between generators and discriminators is not easily realized. Zhu et al. [42] utilized Transformer Prediction Heads (TPH) to globally model the feature maps. While it enhances the detection capabilities for small objects, the complex structure of the Transformer also introduces a certain degree of redundancy to the model. In addition to the problem of small object detection, the number of feature channels can be directly reduced by the feature map extraction process using a 1 × 1 convolution, potentially leading to the loss of feature channel information. At the same time, the detection accuracy can be affected by insufficient feature fusion. Therefore, a traffic sign detection model, YOLOv7-Traffic Sign (YOLOv7-TS), based on sub-pixel convolution [43] and feature fusion is proposed in this study. Firstly, the up-sampling capability of sub-pixel convolution is utilized to integrate the channel dimension pixels and preserve channel information, thus extracting feature maps with richer features. Additionally, a small object detection layer is introduced to the feature fusion network, and the integration map is employed to enhance information interaction among all feature layers, which reduces the rate of small object omissions and improves the effectiveness of feature fusion. Furthermore, the highest-layer feature enhancement is accomplished by the addition of global average pooling and global max pooling, which provide the highest-layer feature with global information and significant information. Feature fusion is then employed to impart the benefits of the highest-layer feature to every other layer, ultimately enhancing the detection accuracy of the model. The major contributions of this study can be summarized as follows:

1. A Feature Map Extraction Module (FMEM) is devised to mitigate channel information loss during feature map extraction by employing sub-pixel convolution.
2. A Multi-feature Interactive Fusion Network (MIFNet) is developed to leverage the rich details of the small object detection layer and employ the integration map to enhance the interaction of information across all feature layers. The perceivability for small traffic sign objects is enhanced, and the effect of feature fusion is improved while reducing the model parameters.
3. A Deep Feature Enhanced Module (DFEM) is established, in which the highest feature is enriched by integrating global average pooling and global max pooling within the Spatial Pyramid Pooling Cross-Stage Partial Channel (SPPCSPC). Simultaneously, pooling is accelerated without altering the size of the original receptive field, thereby speeding up the inference of DFEM.
4. To validate the effectiveness of YOLOv7-TS, a substantial number of experiments are conducted on the CCTSDB2021 [44] and TT100K traffic sign datasets. These experiments demonstrate the commendable detection performance of YOLOv7-TS.

## 2. Related Work

### 2.1. YOLOv7

**Figure 1:**
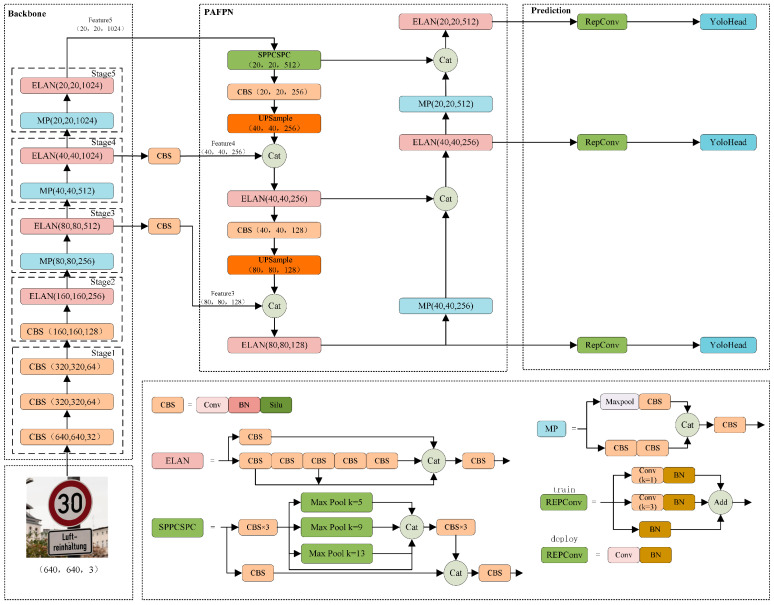
Overall architecture of YOLOv7.

The latest object detector in the YOLO series is YOLOv7 [45], and the structure is depicted in Figure 1. It primarily consists of four components, namely, input, backbone, feature fusion network, and prediction. The complete YOLOv7 detection process is as follows: Initially, the images are preprocessed by YOLOv7, resizing them to a fixed size of 640 × 640 × 3 pixels to meet the input requirements of the backbone. Subsequently, deeper features are continuously extracted within the backbone using CBS modules, Efficient Long-range Attention Network (ELAN) [46] modules, and Max Pooling Reduction (MP) modules. Among these components, CBS comprises the convolution, batch normalization, and Sigmoid Linear Units (SiLU) activation function. The ELAN is constructed by stacking different CBS modules, employing a denser residual structure to aid optimization. The four feature layers, each processed through the different CBS modules, are stacked and subsequently integrated through another CBS module. This integrated result in the output feature has twice the number of channels as the input feature. The MP module employs max pooling in the upper branch and convolution in the lower branch. In both branches, the number of channels is halved by a 1 × 1 convolution, and both branches are spliced to produce down-sampled output feature with the same number of channels as the input feature. The YOLOv7 backbone outputs three feature layers of different sizes: 20 × 20 × 1024, 40 × 40 × 1024, and 80 × 80 × 512. These feature layers are then subjected to channel number adjustment through a 1 × 1 convolution and subsequently input into the Path Aggregation FPN (PAFPN) for feature fusion. The optimization process is aided by the SPPCSPC module, which leverages residual edges while enabling the highest feature to acquire receptive field information at different scales. The number of channels of the three feature layers of varying sizes is adjusted using RepConv, which draws inspiration from the concept of Re-parameterization Visual Geometry Group (RepVGG) [47] and introduces three residual branches to assist in training. These channel-adjusted feature layers are then fed into the YoloHead for prediction. In the deployment phase, the intricate residual structure is reparameterized into a 3 × 3 convolution, reducing the network complexity while maintaining high prediction performance. YOLOv7 outperforms all known object detection models in both speed and accuracy, ranging from 5 to 160 Frames Per Second (FPS). Its exceptional performance makes it suitable to be used as a baseline model for traffic sign detection. However, issues such as the loss of channel information and detection leakage of small traffic sign objects still exist. Therefore, this study focuses on improving YOLOv7 to create an object detector specifically tailored for traffic sign detection.

### 2.2. Sub-Pixel Convolution

**Figure 2:**
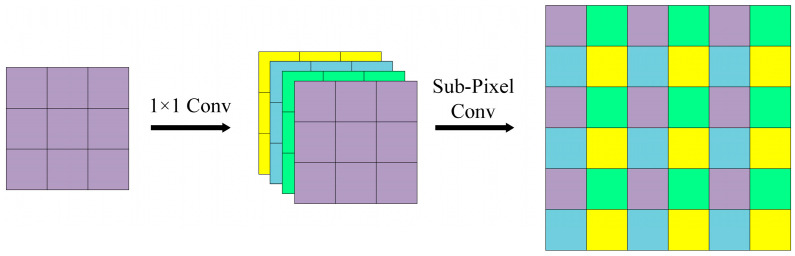
The process of sub-pixel convolution up-sampling.

Sub-pixel convolution is proposed for image super-resolution tasks, in which low-resolution images are super-resolved by generating high-resolution images through sub-pixel convolution. Therefore, sub-pixel convolution is an up-sampling method, and its sampling process is shown in Figure 2. For the input image *W × H × C* (where *W*, *H*, and *C* refer to the width, height, and channel dimensions, respectively), the number of image channels is firstly expanded to *C × r*^2^ with a 1 × 1 convolution, where *r* is the expansion factor. The expanded image becomes *W × H × C × r*^2^. Subsequently, the pixels in *C* are shuffled by the sub-pixel convolution to enlarge *W* and *H* by *r* times and obtain the up-sampled image, *r W × r H × C*. Mathematically, the pixel shuffle operator, *PS*, can be expressed as:

(1) $$PS{\left(T\right)}_{x,y,c}=T\left\lfloor x/r\right\rfloor ,\left\lfloor y/r\right\rfloor ,C\cdot r\cdot \mathrm{mod}\left(y,r\right)+C\cdot \mathrm{mod}\left(x,r\right)+c$$

where *T* denotes the input feature and *PS*(*T*)*_x,y,c_* denotes the output feature pixels at the (*x*, *y*, *c*) coordinates. By initially adjusting the number of channels and subsequently applying sub-pixel convolution, it is ensured that the number of channels of the output image is consistent with that of the input image, while simultaneously increasing the resolution.

### 2.3. Integration Map

A variety of semantic information is contained within the various feature layers of a neural network. Rich semantic information is encompassed by deep features, which is utilized for recognizing object types; rich detail information is abundant in shallow features, which is utilized to clearly localize objects. Due to their high-resolution, small objects can be more effectively perceived using shallow features. The fusion of both deep and shallow features is common in digital image processing tasks. Fan et al. [48] achieved good performance by employing a feature fusion module to receive and process different levels of features. An effective framework was established using FPN for merging feature maps at different scales to accomplish visual tasks through a top-down pathway, which has subsequently been widely adopted and subjected to extensive research [49,50]. An additional bottom-up pathway has been explored using PAFPN to further enhance the low-level information in the deep layers. A weighted bidirectional FPN has been proposed by Scalable and Efficient Object Detection (EfficientDet) [51] for executing simple and rapid feature fusion. Nevertheless, the fused features are required to possess balanced information from each level of resolution. The fused features are influenced by the aforementioned methods, placing greater emphasis on neighboring resolutions and reducing the significance of features with farther resolutions. As a result, the semantic information within the non-neighboring layers becomes diluted during the continuous feature fusion.

**Figure 3:**
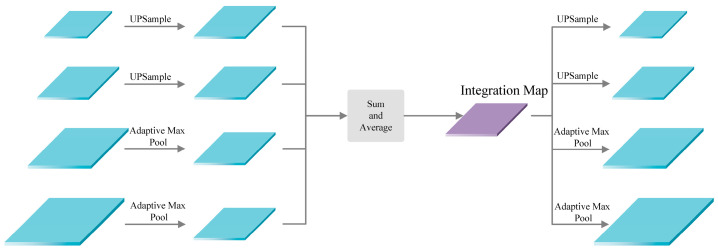
The application process of integration map.

Integrating and refining multi-scale features through the introduction of a balanced feature pyramid is achieved by Libra R-CNN. The main structure utilized for this purpose is the integration map, which serves to enhance the interaction of information between all feature layers and mitigate the loss of semantic information in non-adjacent feature layers during the fusion process. The application process of the integration map is depicted in Figure 3. The low-resolution features are subjected to up-sampling through linear interpolation, while the high-resolution features are adjusted to the same resolution size via adaptive max pooling. Subsequently, the integration map is generated through simple averaging. At this point, rich information in multi-scale feature layers is incorporated into the integration map, with due consideration given to the information from non-adjacent layers. Following this, the restoration of the original resolution features is facilitated by the integration map through linear interpolation up-sampling and adaptive max pooling, enhancing the original features across all layers.

## 3. YOLOv7-Traffic Sign (YOLOv7-TS)

### 3.1. Overall Architecture

**Figure 4:**
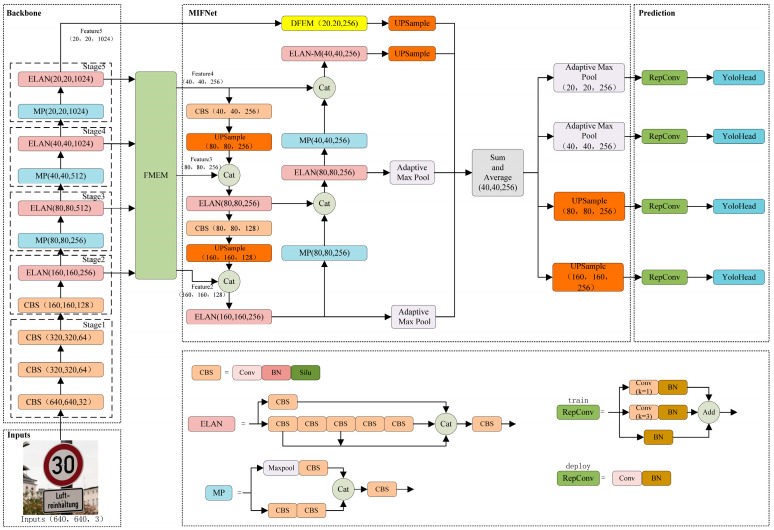
The architecture of our proposed YOLOv7-TS.

While traffic sign detection algorithms based on the YOLO series have indeed achieved remarkable results in terms of both detection accuracy and speed, they still encounter three significant challenges. The first issue pertains to the loss of channel information during the feature map extraction process, while the second relates to the high detection leakage rate of small objects. The third issue concerns both the loss of information in the fusion process of non-adjacent feature layers and the inadequacy of the fusion. To address these issues, YOLOv7-TS is proposed as an extension of YOLOv7, and its structure is depicted in Figure 4. The YOLOv7-TS primarily comprises four components: input, backbone, feature fusion network, and prediction. The backbone is employed to extract features at various resolutions. The Feature Map Extraction Module (FMEM) is positioned between the backbone and the feature fusion network to facilitate feature map extraction, ensuring the retention of rich channel information from different feature layers. The feature maps extracted by FMEM contain both high-level semantic information and low-level detail information, which has a positive role in promoting target recognition and localization. Within the feature fusion network, the Multi-feature Interactive Fusion Network (MIFNet) is utilized to facilitate information interaction among all features, thereby enhancing the effectiveness of feature fusion. Simultaneously, the integration of detailed information from the high-resolution feature map aids in the precise localization of small objects, mitigating the issue of missed detections for small objects. Additionally, the highest-layer feature is enhanced using the Deep Feature Enhanced Module (DFEM), ensuring the provision of both global and significant information, thereby benefiting all layers through feature fusion. Ultimately, the fused features are subjected to classification and regression within the final prediction component.

The complete process for YOLOv7-TS is as follows: The input images are initially adjusted to a fixed size of 640 × 640 × 3 and fed into the YOLOv7-TS backbone. Then, four feature layers, *S*_2_, *S*_3_, *S*_4_, and *S*_5_, are generated by the backbone, corresponding to the outputs of stages 2 to 5, with 256, 512, 1024, and 1024 channels, respectively. The output, *S*_2_, of stage 2 is added as the small object detection layer. Subsequently, FMEM performs the sub-pixel convolution for the inputs *S*_2–5_ to transform the channel dimension information of the different feature layers into spatial dimensions. The up-sampled images are then added element-by-element, with the initial feature layers of the same resolution, to obtain the feature maps *F*_2–4_. The feature map *F*_5_ is generated directly from *S*_5_ without any other operations. Further, *F*_2–5_ are processed by MIFNet for feature fusion, where *F*_2–4_ are channeled into PAFPN for pyramid feature fusion, while *F*_5_ is directly involved in the generation of the integration map after feature enhancement and no longer enters PAFPN. The purpose of this step is to alleviate the increase in the number of parameters brought about by the four-layer feature fusion, and simultaneously, avoid excessive feature fusion that could potentially affect the fusion effect. The DFEM is used to perform feature enhancement for *F*_5_. Building upon the original SPPCSPC, global average pooling and global max pooling are incorporated in the residual path to provide global information and significant information to the highest-layer feature, respectively. Simultaneously, the original pooling structure is altered by DFEM, and pooling continues based on the result of the previous layer using a small pooling kernel. This achieves pooling acceleration with the same receptive field to enhance the overall module speed. The feature-enhanced *F*_5_ and the pyramidal feature-fused *F*_2–4_ are jointly involved in generating the integration map for the interaction among all feature layers and fusion effect enhancement. Finally, each feature layer is restored to its original resolution for subsequent predictions by max pooling and linear interpolation up-sampling. The details and design philosophy of this network will be elaborated in Section 3.2, Section 3.3 and Section 3.4.

### 3.2. Feature Map Extraction Module (FMEM)

**Figure 5:**
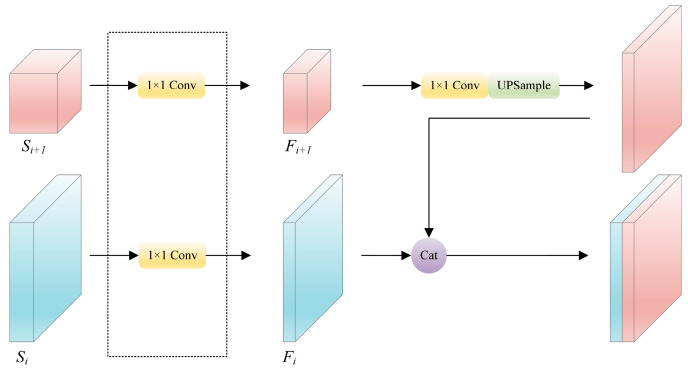
The process of using 1 × 1 convolution to extract specific channel number features in YOLO series algorithms.

As representatives of the One-Stage object detection algorithms, the YOLO series algorithms have been widely employed for real-time detection due to their exceptional detection speed. In pursuit of computational efficiency, the YOLO series algorithms employ a simple 1 × 1 convolution to extract features with a specific number of channels for feature fusion, as demonstrated in Figure 5. However, this practice results in the loss of channel information, even with the latest iteration, i.e., YOLOv7. Recently, numerous FPN-based networks have been predominantly applied for the development of efficient modules within the pyramid to enhance fusion results. However, these approaches do not inherently tackle the issue of channel information loss and fail to effectively harness the rich channel information present in the backbone output features.

**Figure 6:**
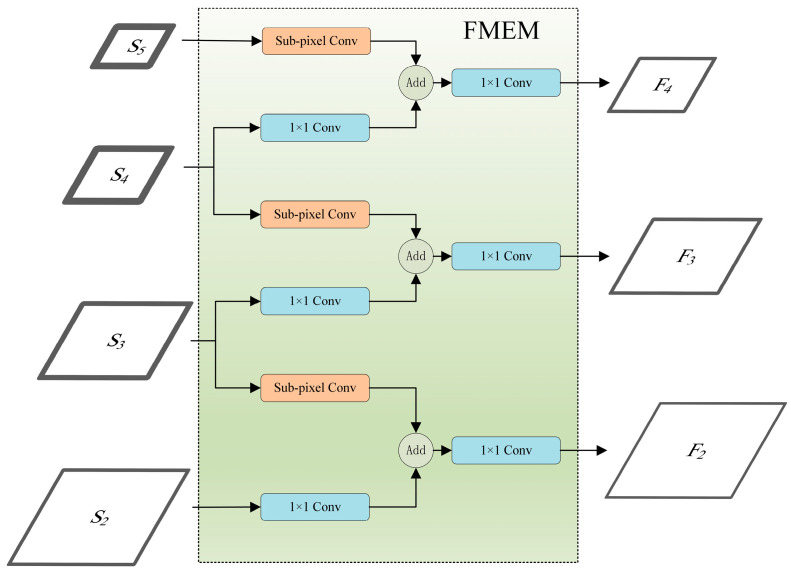
The structure of the proposed FMEM.

The FMEM is designed to solve this issue by leveraging the abundant channel information within *S_i_* through sub-pixel convolutional up-sampling, ultimately enhancing the detection capabilities. Sub-pixel convolution, as a form of up-sampling, contributes to the model in the following aspects: Firstly, it aids in recovering the lost detailed information in deep features while preserving rich semantic information within channels. Secondly, the feature maps extracted through sub-pixel convolution encompass both high-level semantic information and low-level detailed information, actively facilitating simultaneous object localization and recognition. Thirdly, by increasing the spatial dimension, the model fully learns the positional information of small objects, resulting in a more precise localization of the bounding boxes for small objects. This up-sampling method firstly requires the image to expand the number of channels by a 1 × 1 convolution, and then, increase the spatial dimension by shuffling the channel dimension pixels. The feature channel information is not lost, but transformed into spatial dimension information and retained. Typically, additional computational overhead is introduced by the process of expanding the number of channels. However, the sufficient number of channels of *S*_3–5_ in this study allows the images to be up-sampled directly using a sub-pixel convolution, and the need to expand the number of channels using a 1 × 1 convolution is nullified. Therefore, the feature channel information is retained without requiring additional computation. The structure of FMEM is shown in Figure 6. Once *S*_2–5_ are input into the FMEM, *S*_2_ is solely involved in channel adjustment through a 1 × 1 convolution; *S*_5_ exclusively employs sub-pixel convolution; whereas *S*_3_ and *S*_4_ simultaneously engage in sub-pixel and 1 × 1 convolutions. Subsequently, the features obtained through sub-pixel convolution and channel number adjustment are added element-wise, followed by a 1 × 1 convolution, resulting in feature maps *F*_2–4_. The *F*_2–4_ feature maps are generated by fusing the rich channel information in *S*_3–5_, based on *S*_2–4_. The FMEM process can be expressed using the following equations:

(2) $$F_{2}=C^{1\times 1}\left(C^{1\times 1}\left(S_{2}\right)+SPC\left(S_{3}\right)\right)$$

(3) $$F_{3}=C^{1\times 1}\left(C^{1\times 1}\left(S_{3}\right)+SPC\left(S_{4}\right)\right)$$

(4) $$F_{4}=C^{1\times 1}\left(C^{1\times 1}\left(S_{4}\right)+SPC\left(S_{5}\right)\right)$$

where *S_i_* and *F_i_* denote the input and output of FMEM, *C*^1×1^ represents the 1 × 1 convolution, and *SPC* is the sub-pixel convolution. Subsequently, the obtained feature maps are input into MIFNet for feature fusion.

### 3.3. Multi-Feature Interactive Fusion Network (MIFNet)

Traffic sign detection mainly occurs in autonomous driving and assisted driving scenarios, and there are more small objects at a distance. This is primarily because there is a braking distance following emergency braking maneuvers, and early detection of traffic signs is essential to alert the driver and mitigate potential risks. YOLOv7 possesses the detection speed and model size requirements for traffic sign detection scenarios. However, the down-sampling multiple of YOLOv7 is relatively large, feature maps with the resolution of 20 × 20, 40 × 40, 80 × 80 in the lower three layers are usually selected, and it is difficult for the deeper feature maps to learn the features of small objects. Furthermore, YOLOv7 utilizes the PAFPN structure for feature fusion, merging semantic information from higher levels to naturally impart distinct contextual information to lower-level feature maps. Nonetheless, the feature fusion process exclusively involves the combination of neighboring feature layers, neglecting direct interactions of non-neighboring feature layers, and diluting their semantic information over multiple iterations.

**Figure 7:**
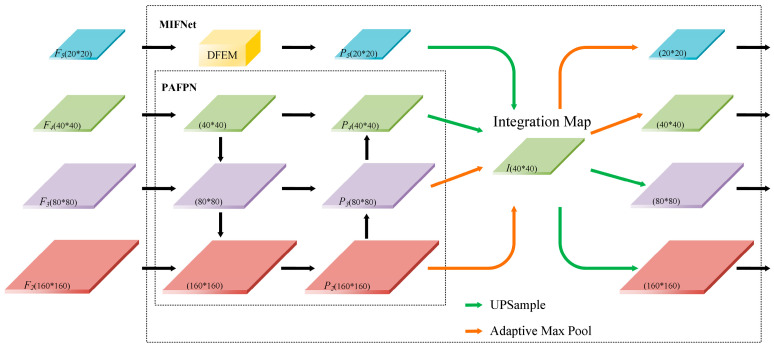
The structure of the proposed MIFNet.

In this study, we establish MIFNet to improve the detection performance of YOLOv7 for small objects and facilitate information interaction across all feature layers. Firstly, four feature layers are fed as inputs to MIFNet. Incorporating a high-resolution feature map to provide additional detailed information and employing feature fusion benefits the overall model, thus enhancing the perception of small objects. Inspired by Libra R-CNN, we further combine the integration map with PAFPN to augment multi-level features using the same depth-integrated balanced semantic feature map to enhance the information interaction between all feature layers. Lastly, to reduce the model parameters, the highest-level feature is directly utilized for integration map generation after feature enhancement, without utilizing the pyramid feature fusion of PAFPN. The rich contextual information in the highest-level feature is not lost but fully harnessed in generating the integration map, benefiting the other layers. Moreover, when the highest-level feature is fully utilized, excessive fusion can lead to unnecessary aliasing effects, which can have a negative impact on the fusion results. The structure of MIFNet is illustrated in Figure 7, where *F*_3–5_ are the original input feature layers and *F*_2_ is the added small object detection layer. PAFPN is utilized to fuse the features *F*_2–4_ to obtain the *P*_2–4_ feature layers with increased perception of small objects. Additionally, *F*_5_ alone uses the DFEM for feature enhancement to generate feature *P*_5_, and the details of this module are described in Section 3.4. The integration map, *I* (40 × 40 × 256; consistent with *F*_4_), is created from the four feature layers after pyramid feature fusion or enhancement by linear interpolation up-sampling and adaptive max pooling, respectively. The information interaction between features at different scales is facilitated by this process, thus ensuring the full utilization of features at each layer. The process of generating an integration map can be given by the following equation:

(5) $$C_{2}=MaxPool\left(P_{2}\right)$$

(6) $$C_{3}=MaxPool\left(P_{3}\right)$$

(7) $$C_{4}=UPSample\left(P_{4}\right)$$

(8) $$C_{5}=UPSample\left(P_{5}\right)$$

(9) $$I=\frac{1}{4}\sum_{i=2}^{5}C_{i}$$

where *P_i_* denotes the features generated from *S_i_* after PAFPN feature fusion or DFEM feature enhancement, *C_i_* represents the result of *P_i_* after max pooling or up-sampling, *MaxPool* stands for the adaptive max pooling operation, and *UPSample* denotes linear interpolation up-sampling. The feature layers are then restored to the original resolution by adaptive max pooling and linear interpolation up-sampling for subsequent prediction.

### 3.4. Deep Feature Enhanced Module (DFEM)

**Figure 8:**
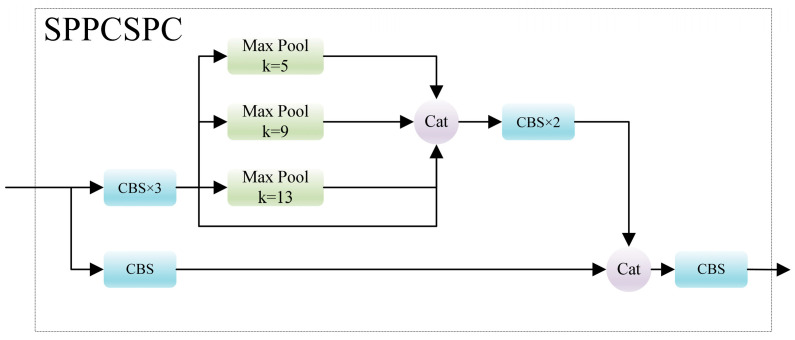
The structure of SPPCSPC.

Widely employed for enhancing the contextual information of features, SPP entails the extraction of various receptive field features to output fixed-size feature vectors. In YOLOv7, the SPPCSPC structure imparts distinct contextual information to the highest-level feature, enhancing every feature layer through the feature pyramid. The structure of SPPCSPC is illustrated in Figure 8. In the SPPCSPC structure, three independent pooling layers with varying kernels are employed to capture the contextual information from different receptive fields in the highest-level feature. The entire process can be represented by the following equation:

(10) $$X=C^{1\times 1}\left(C^{3\times 3}\left(C^{1\times 1}\left(F_{5}\right)\right)\right)$$

(11) $$R_{1}=MaxPool^{k=5}\left(X\right)$$

(12) $$R_{2}=MaxPool^{k=9}\left(X\right)$$

(13) $$R_{3}=MaxPool^{k=13}\left(X\right)$$

(14) $$P5=C^{1\times 1}\left(Cat\left(C^{3\times 3}\left(C^{1\times 1}\left(Cat\left(R_{i},X\right)\right)\right),C^{1\times 1}\left(F_{5}\right)\right)\right),i=1,2,3$$

where *F*_5_ denotes the input feature layer, *X* represents the feature that will undergo pooling operations, *C*^1×1^ stands for the 1 × 1 convolution, *C*^3×3^ denotes the 3 × 3 convolution, *k* is the size of the max pooling kernel, *R_i_* signifies the result after max pooling, *P*_5_ symbolizes the total output of SPPCSPC, and *Cat* is the concatenation operation.

**Figure 9:**
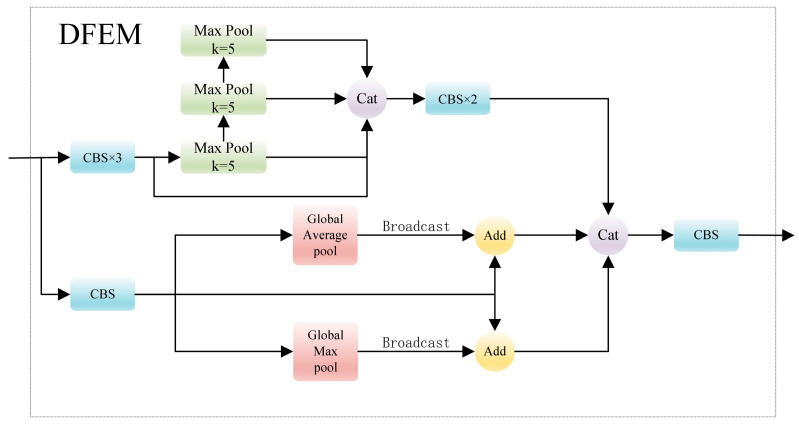
The structure of the proposed DFEM.

To enhance the highest-level feature and improve the fusion effect of MIFNet, DFEM is introduced on the basis of SPPCSPC in this study, as illustrated in Figure 9. In the separate residual path, global average pooling and global max pooling are introduced initially. The outcomes of these pooling operations are subsequently broadcast to align with the resolution of the feature in the residual branch and added element-by-element to enhance the original feature. Simultaneously, inspired by SPPF in YOLOv5, the three independent pooling layers are improved in DFEM by connecting them in a way that small pooling kernel layers are utilized for output. This approach not only achieves pooling results equivalent to those obtained with a larger pooling kernel, preserving the receptive field, but also facilitates accelerated pooling, thereby enhancing the overall inference speed of the entire module. Ultimately, the result from the residual branch is concatenated with the accelerated pooling result to obtain the final output. This process can be represented using the following equations:

(15) $$X=C^{1\times 1}\left(C^{3\times 3}\left(C^{1\times 1}\left(F_{5}\right)\right)\right)$$

(16) $$R_{1}=MaxPool^{k=5}\left(X\right)$$

(17) $$R_{2}=MaxPool^{k=5}\left(R_{1}\right)$$

(18) $$R_{3}=MaxPool^{k=5}\left(R_{2}\right)$$

(19) $$Y=C^{1\times 1}\left(F_{5}\right)$$

(20) $$P5=C^{1\times 1}\left(Cat\left(C^{3\times 3}\left(C^{1\times 1}\left(Cat\left(R_{i},X\right)\right)\right),\theta \left(GMP\left(Y\right)\right)+Y,\theta \left(GAP\left(Y\right)\right)+Y\right)\right),i=1,2,3$$

where *F*_5_ denotes the input feature layer, *X* represents the feature to undergo the max pooling operation, *C*^1×1^ stands for the 1 × 1 convolution, *C*^3×3^ stands for the 3 × 3 convolution, *k* means the max pooling kernel size, *R_i_* represents the results after max pooling, *Y* represents the feature that will undergo global pooling, *GMP* signifies the global max pooling, *GAP* signifies the global average pooling, *θ* symbolizes the broadcasting operation, *P*_5_ manifests the total output of DFEM, and *Cat* is the concatenation operation. Through DFEM, the overall pooling process is accelerated while retaining the original receptive field. Simultaneously, the original feature layer is endowed with global information and crucial information, enhancing the overall features.

## 4. Experiments and Discussion

To demonstrate the effectiveness of the proposed model in this study, the experimental results are discussed in this section. To start, the datasets used in the experiments are introduced. Following this, a comprehensive presentation of the experiment setup and evaluation criteria is provided. Subsequently, we discuss the ablation experiments conducted to validate the effectiveness of the introduced modules. Finally, to verify the advantages of the proposed model, the model is compared with the baseline model and other common models.

### 4.1. Datasets

#### 4.1.1. CCTSDB2021

**Figure 10:**
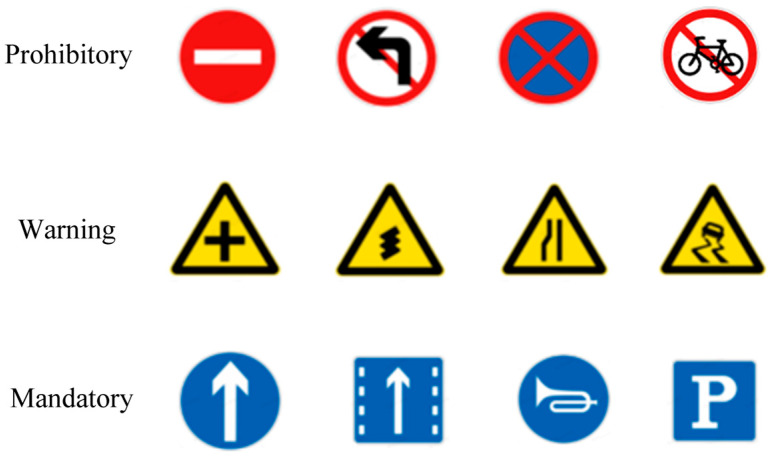
Sample traffic signs of the three categories in the CCTSDB2021 dataset.

**Figure 11:**
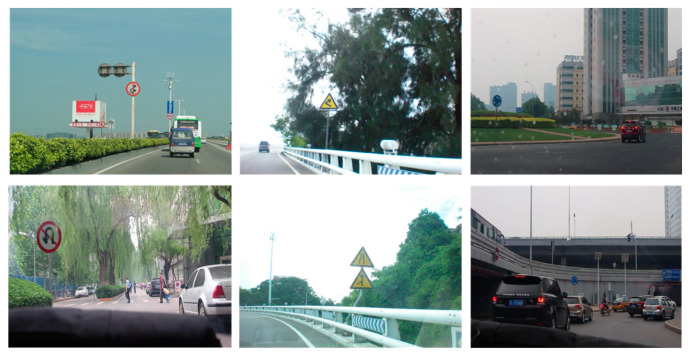
Sample images of the CCTSDB2021 dataset.

The CCTSDB2021 dataset, created by Changsha University of Science and Technology, China, is one of the most recognized traffic sign datasets. The dataset is subdivided into three important categories, namely, “mandatory”, “prohibitory”, and “warning”. The training and validation sets consist of 16,356 and 1500 images, respectively. To enrich the training dataset, images captured under a variety of weather conditions, such as foggy weather and snowy conditions, are also included in the CCTSDB2021 dataset. Examples of the three different categories of traffic signs and sample images from the CCTSDB2021 dataset are illustrated in Figure 10 and Figure 11, respectively.

#### 4.1.2. TT100K

**Figure 12:**
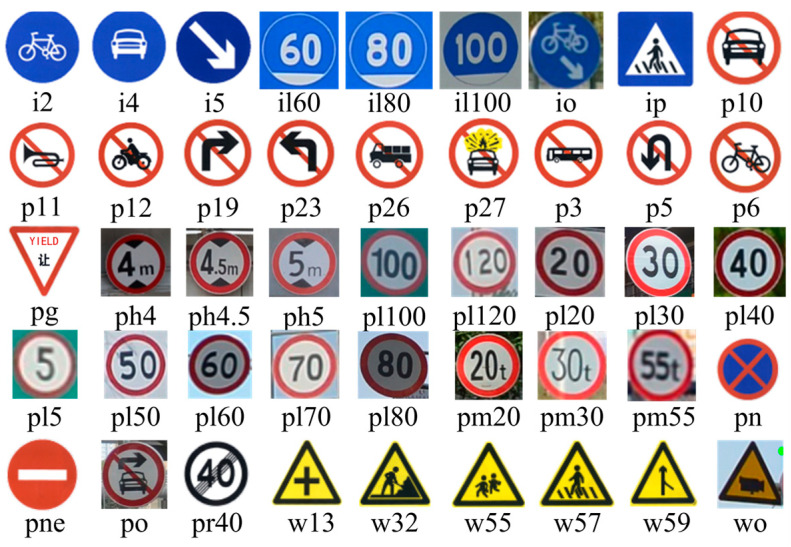
The 45 traffic sign classes with more than 100 instances in the TT100K dataset.

**Figure 13:**
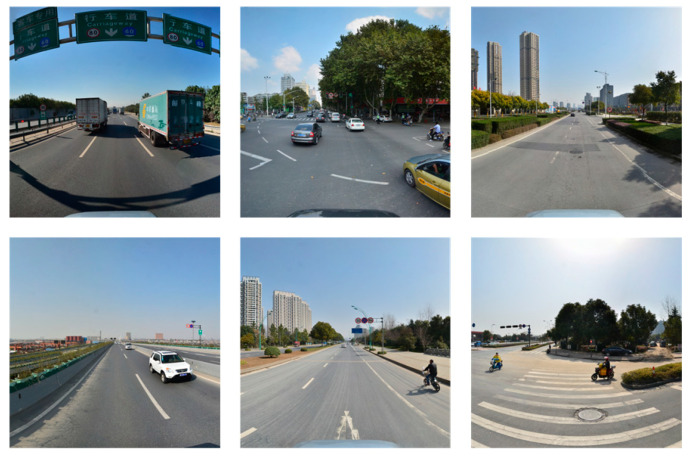
Sample images of the TT100K dataset.

The TT100K dataset, curated and released by a joint laboratory between Tsinghua University and Tencent, comprises ~100,000 street-level images from various cities in China, and the traffic signs in these images are annotated with bounding boxes. A comprehensive range of traffic sign categories is covered by the dataset, totaling 221 different classes of traffic signs, comprising a total of 9176 images. The training and validation sets comprise 6105 and 3071 images, respectively. A significant data imbalance issue is encountered due to only 45 classes having over 100 instances and nearly half of the classes having only single-digit instances. Consequently, for our experiments, only these 45 traffic sign categories are selected, and all other classes in the TT100K dataset are excluded. The training and validation sets are subsequently reconfigured to a 9:1 ratio. The 45 traffic sign classes with over 100 instances in the TT100K dataset and sample images from the TT100K dataset are shown in Figure 12 and Figure 13.

### 4.2. Evaluation Metrics and Implementation Details

#### 4.2.1. Evaluation Metrics

To quantitatively evaluate the performance of proposed model, precision (P), recall (R), F1 score (F1), mean average precision (mAP), the number of parameters (Params), and frames per second (FPS) are adopted as the evaluation metrics. The proportion of true positive samples among samples detected as positive by the model is denoted as P. R represents the proportion of true positive samples detected by the model among all positive samples. F1 combines both precision and recall to reflect the model’s detection performance, with a higher value indicating better detection performance. The mAP is the average value of AP for all categories, where AP means the accuracy for a single class. A higher mAP value indicates better overall detection accuracy for the model. Params is employed to measure the model’s complexity, and it is related to the amount of computer memory resources the model occupies. Smaller Params values indicate fewer model parameters and less memory usage. FPS is the number of images the model can detect per second. A higher FPS indicates faster model speed and the ability to process a larger number of images per second. These evaluation metrics can be calculated using the following formulas:

(21) $$P=\frac{TP}{TP+FP}$$

(22) $$R=\frac{TP}{TP+FN}$$

(23) $$F1=\frac{2PR}{P+R}$$

(24) $$AP=\int_{0}^{1}P(R)dR$$

(25) $$mAP=\frac{1}{N}\sum_{i=1}^{N}AP_{i}$$

where *TP* represents the number of samples correctly detected as traffic signs, *FP* means the number of detected traffic signs with incorrect labels compared with the ground truth, *FN* denotes the number of samples where traffic signs are present in reality but not detected, and *N* is the total number of detected categories.

#### 4.2.2. Implementation Details

To maintain objectivity, all the experiments in this study are performed on a setup with the following specifications: Linux operating system; PyTorch 1.7.1, CUDA version 11.7; and NVIDIA A100 40 G GPU. The reported FPS values in this study are obtained directly in the GPU environment. The IoU threshold for calculating mAP is set to 0.5. We employe the Adam training optimizer with an initial learning rate of 0.001. To save memory space, the batch size is set to 8. During training, the number of epochs is set to 100 and 300 for the CCTSDB2021 and TT100K datasets, respectively.

### 4.3. Ablation Experiments

To confirm the effectiveness of the modules proposed in YOLOv7-TS, extensive ablation experiments are conducted on both datasets and the experimental results are analyzed.

#### 4.3.1. Ablation Study of FMEM

With YOLOv7 adopted as the baseline model, the FMEM input feature layer is modified to include three layers to match the three-layer output of the YOLOv7 backbone. FMEM is then incorporated into YOLOv7 to extract feature maps while reducing channel number loss. The specific experimental results of these two methods on the CCTSDB2021 and TT100K datasets are displayed in Table 1.

YOLOv7 obtains the values of 92.11%, 70.45%, 79.84%, and 82.39% for P, R, F1, and mAP, respectively, for the CCTSDB2021 dataset. However, after addressing the channel information loss, YOLOv7 + FMEM achieves P, R, F1, and mAP values of 93.12%, 72.69%, 81.65%, and 83.70%, respectively, which are 1.01%, 2.24%, 1.81% and 1.31% higher than the respective values for YOLOv7. Similarly, on the TT100K dataset, YOLOv7 + FMEM achieves P, R, F1, and mAP increments of 0.32%, 1.16%, 0.78%, and 0.94%, respectively, compared with YOLOv7. This comparative analysis of experimental data for the two methods demonstrates the superior detection performance and accuracy of YOLOv7 + FMEM. This effect is due to a reduction in the channel information loss by FMEM during feature map extraction, improving the overall detection performance.

**Figure 14:**
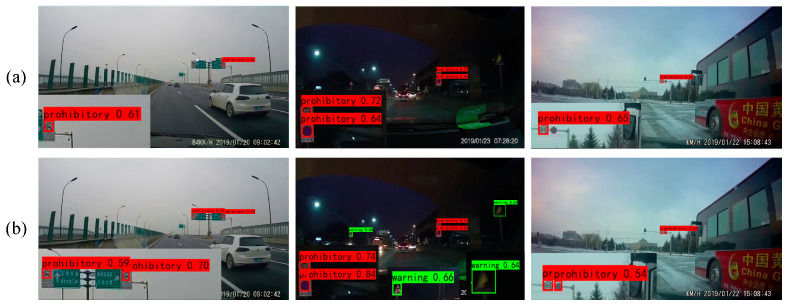
Ablation results for FMEM tested on the CCTSDB2021 dataset. (**a**) YOLOv7; (**b**) YOLOv7 + FMEM.

**Figure 15:**
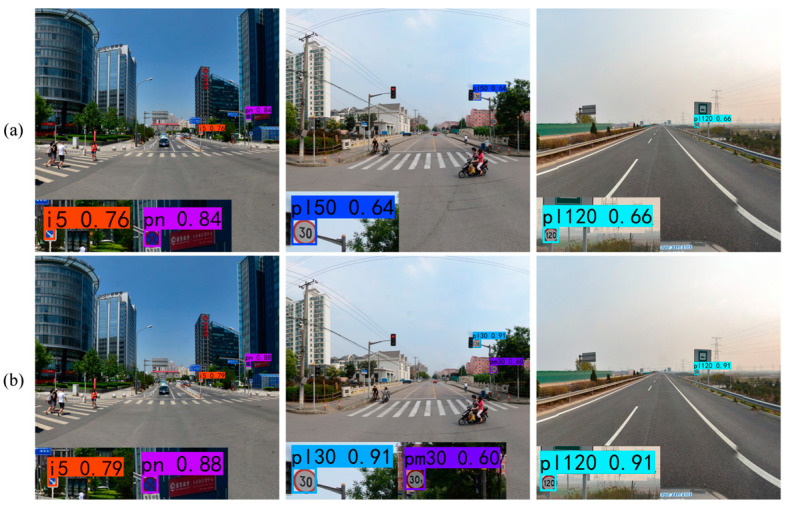
Ablation results for FMEM tested on the TT100K dataset. (**a**) YOLOv7; (**b**) YOLOv7 + FMEM.

To present the results of the ablation experiments more clearly, examples of the results with both datasets are provided for the two methods in Figure 14 and Figure 15. From these figures, it is evident that YOLOv7 detects objects with relatively low confidence scores. However, after incorporating FMEM, the confidence scores for all detected objects increase due to its ability to preserve the channel information lost in basic YOLOv7. Among the objects missed by YOLOv7, some are detected after the addition of FMEM, but a few individual objects remain undetected, which indicates that while the preserved channel information may not completely resolve the issue of missing small objects, it still has a positive impact. Meanwhile, in the second set of comparison images in Figure 14, YOLOv7 misclassifies a “speed limit 30” traffic sign as “speed limit 50”, which is corrected upon FMEM incorporation. In summary, these experimental results collectively provide evidence of the effectiveness of FMEM.

#### 4.3.2. Ablation Study of MIFNet

The impact of the MIFNet on traffic sign detection performance is also investigated. Ablation experiments are conducted on both datasets to assess the following models: (a) baseline YOLOv7, (b) YOLOv7 + integration map, (c) YOLOv7 + integration map + small object detection layer, and (d) YOLOv7 + MIFNet. The experimental results for these four methods are presented in Table 2.

Using the CCTSDB2021 dataset, YOLOv7 + integration map achieves P, R, F1, and mAP values of 93.87%, 71.89%, 81.42%, and 82.99%, respectively, demonstrating an improvement of 1.76%, 1.44%, 1.58%, and 0.60% compared with YOLOv7. This improvement can be attributed to the enhanced feature fusion effect due to the substantial information interaction among all feature layers. Further, with the addition of the small object detection layer, the obtained values are 90.34%, 78.21%, 83.84%, and 83.91%, respectively. While the P value is slightly lower than that of YOLOv7, the R, F1, and mAP values exhibited improvements of 7.76%, 4.00%, and 1.52%, respectively. This indicates that the high-resolution feature provides more detailed information, facilitating the model recognition of small objects. Finally, YOLOv7 + MIFNet achieves P, R, F1, and mAP values of 92.23%, 78.36%, 84.73%, and 84.02%, respectively, with the CCTSDB2021 dataset, demonstrating an improvement of 0.12%, 7.91%, 4.89%, and 1.63%, respectively, compared with YOLOv7, and 1.89%, 0.15%, 0.89%, and 0.11%, respectively, compared with YOLOv7 + integration map + small object detection layer. Although the number of feature layers used for the pyramid feature fusion is reduced with MIFNet, the detection accuracy does not decrease, but rather shows a slight improvement. This indicates that when feature information is already fully utilized, additional rounds of feature fusion may not necessarily result in performance gains and may even exacerbate model parameter redundancy. Using the TT100K dataset, excellent performance is also demonstrated by YOLOv7 + MIFNet, and all indicators are improved, verifying the superiority of MIFNet for small object detection and feature fusion.

**Figure 16:**
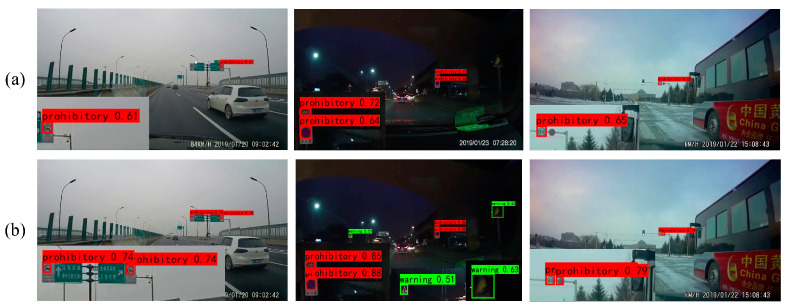
Ablation results for MIFNet tested on the CCTSDB2021 dataset. (**a**) YOLOv7; (**b**) YOLOv7 + MIFNet.

**Figure 17:**
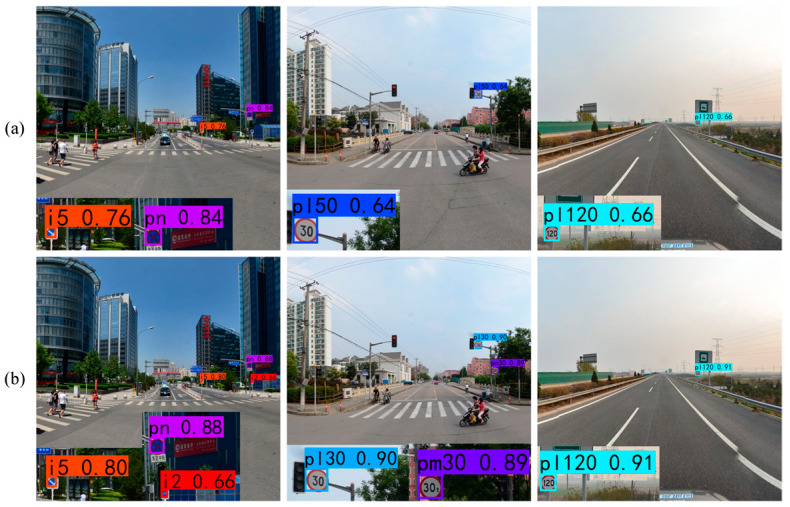
Ablation results for MIFNet tested on the TT100K dataset. (**a**) YOLOv7; (**b**) YOLOv7 + MIFNet.

The comparative sample results of YOLOv7 and YOLOv7 + MIFNet using both datasets are displayed in Figure 16 and Figure 17. From these figures, it is noticeable that after using MIFNet for feature fusion, the detected objects exhibit higher confidence scores. This can be attributed to the excellent feature fusion capability of MIFNet, which enhances the model detection performance. Additionally, YOLOv7 + MIFNet successfully detects and accurately categorizes the objects that are missed or misclassified by YOLOv7 due to the former’s effective utilization of detailed information in the high-resolution feature layer.

To demonstrate that MIFNet can achieve favorable feature fusion effects, attain higher detection accuracy, and simultaneously reduce the model parameters, we present a parameter comparison in Table 3. With the utilization of the integration map, the model parameters decrease by 1.8 M compared with YOLOv7. Upon the addition of the small object detection layer and using the four-layer structure, the model parameters increase by 1.3 M. When applying MIFNet to YOLOv7, the model parameters reduce to 28.2 M, representing a decrease of 8.7 M compared with YOLOv7. Through the experimental data and visualization of detection results, it is conclusively demonstrated that MIFNet effectively reduces the model parameters while simultaneously enhancing accuracy.

#### 4.3.3. Ablation Study of DFEM

To validate the effectiveness of DFEM, we conduct ablation experiments with both datasets for the YOLOv7 + DFEM model. The experimental results are presented in Table 4.

Applied to the CCTSDB2021 dataset, YOLOv7 + DFEM obtains P, R, F1, and mAP values of 94.24%, 71.23%, 81.14%, and 83.71%, respectively, demonstrating improvements of 2.13%, 0.78%, 1.30%, and 1.32%, respectively, compared with YOLOv7. Similarly, with the TT100K dataset, YOLOv7 + DFEM demonstrates improvements of 0.90%, 1.11%, 1.02%, and 1.19% in the P, R, F1, and mAP values, respectively, compared with YOLOv7. The highest-level feature is enhanced by providing it with global and crucial information. After combining it with the different receptive fields provided by the upper branch pooling, all feature layers benefit from feature fusion.

**Figure 18:**
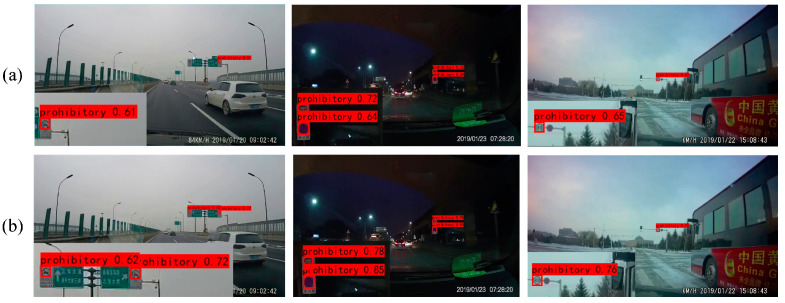
Ablation results for DFEM tested on the CCTSDB2021 dataset. (**a**) YOLOv7; (**b**) YOLOv7 + DFEM.

**Figure 19:**
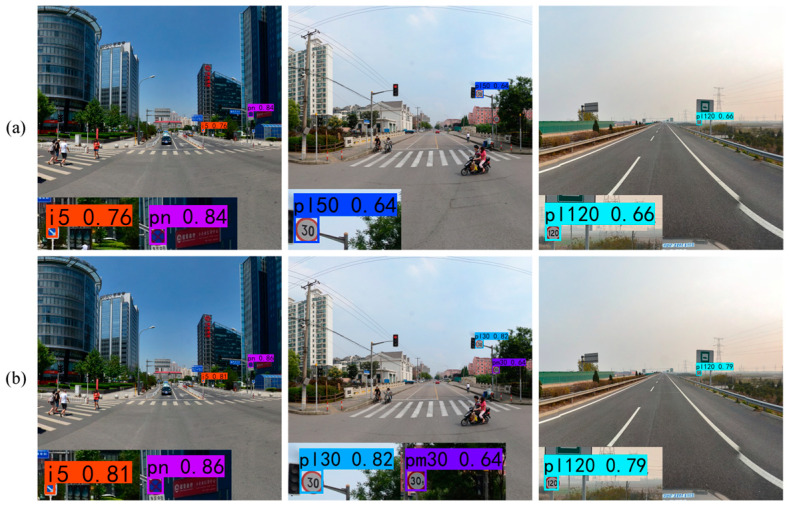
Ablation results for DFEM tested on the TT100K dataset. (**a**) YOLOv7; (**b**) YOLOv7 + DFEM.

The comparative detection results of YOLOv7 and YOLOv7 + DFEM using the CCTSDB2021 and TT100K datasets are shown in Figure 18 and Figure 19. Compared with YOLOv7, the enhancement of the highest-level feature by DFEM improves the model detection performance, and the confidence of all detected objects is improved. Some small objects that are initially missed due to the low confidence scores of YOLOv7 are now detected with higher confidence after feature enhancement with DFEM, indicating its positive impact on small object detection. Additionally, objects incorrectly detected by YOLOv7 are also correctly identified by YOLOv7 + DFEM. These results collectively demonstrate the effectiveness of DFEM in enhancing the highest-layer feature.

To demonstrate that the introduction of pooling acceleration in DFEM can speed up the inference of the whole module, we present a parameter and inference time comparison in Table 5 among SPPCSPC, DFEM (without pooling acceleration), and DFEM. By comparing these, it is evident that DFEM (without pooling acceleration) increases the module parameters compared with SPPCSPC, simultaneously extending the inference time. This is due to the fact that DFEM (without pooling acceleration) adds global max pooling and global average pooling to the residual branch on the basis of SPPCSPC. After introducing pooling acceleration in DFEM, the module parameters remain unchanged compared with DFEM (without pooling acceleration). However, the inference time is reduced. Simultaneously, there is a significant improvement in inference speed compared with SPPCSPC.

#### 4.3.4. Ablation Study between Different Modules

The modules proposed in this study achieve varying degrees of performance improvement on the YOLOv7 baseline network. We further conduct ablation experiments to demonstrate that the concurrent application of the three proposed modules on YOLOv7 does not have a negative impact and can yield favorable results, as shown in Table 6.

It can be observed that for the CCTSDB2021 dataset, YOLOv7 + FMEM + MIFNet, which simultaneously focuses on rich channel information and feature fusion, achieves an F1 value of 84.34% and mAP of 85.08%. Values of 82.25% and 84.59%, respectively, are obtained by YOLOv7 + FMEM + DFEM, which benefits from the preservation of channel information and the enhancement of high-level features. YOLOv7 + MIFNet + DFEM, in which high-level features are enhanced and the enhanced features are propagated to other layers through feature fusion, achieved values of 83.96% and 84.83%. Significant improvements in the F1 score and mAP are exhibited by these three methods, indicating that model performance and detection accuracy can be enhanced by any combination of the proposed components in this study. When introducing all the proposed components into YOLOv7 simultaneously, the F1 and mAP values obtained are 84.90% and 86.02%, which are the highest values among the five methods. In addition, the experimental results using TT100K dataset also reach the same conclusion. This ablation experiment fully demonstrates the effectiveness of simultaneously introducing the components proposed in this study into the benchmark model.

### 4.4. Comparison Experiments

To validate the superiority of the YOLOv7-TS model, a comparison is conducted between YOLOv7-TS and the baseline model YOLOv7, as well as other common models.

#### 4.4.1. Comparison Experiment between YOLOv7-TS and YOLOv7

Table 7 presents the comparative experimental results of YOLOv7 and YOLOv7-TS tested on both datasets to demonstrate the superiority of the latter, which is based on the YOLOv7 baseline model and incorporates FMEM, MIFNet, and DFEM.

A noteworthy trend is that YOLOv7-TS makes substantial strides in both model performance and detection accuracy, albeit at the cost of a modest reduction in speed. Specifically, using the CCTSDB2021 dataset, YOLOv7-TS demonstrates improvements of 0.58%, 7.86%, 5.06%, and 3.63% in P, R, F1, and mAP, respectively, compared with YOLOv7. Similarly, with the TT100K dataset, it demonstrates enhancements of 0.90%, 4.59%, 2.89%, and 2.68% in P, R, F1, and mAP, respectively. These advancements can be attributed to the emphasis of YOLOv7-TS on leveraging rich channel information, bolstering feature quality, and refining feature fusion techniques. Moreover, YOLOv7-TS achieves a reduction of 2.2 million parameters relative to YOLOv7, underscoring the efficacy of employing a three-layer feature pyramid fusion approach in MIFNet for mitigating the redundancy of model parameters.

**Figure 20:**
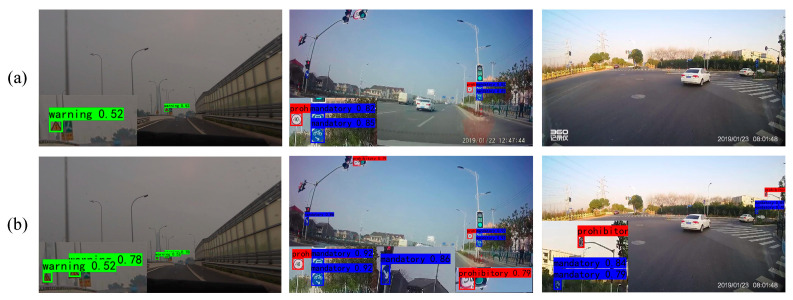
Comparison results of YOLOv7 and YOLOv7-TS tested on the CCTSDB2021 dataset. (**a**) YOLOv7; (**b**) YOLOv7-TS.

**Figure 21:**
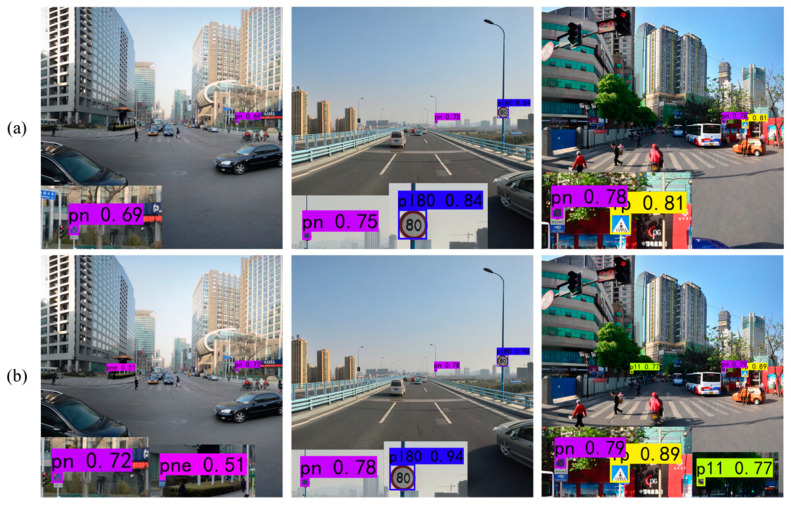
Comparison results of YOLOv7 and YOLOv7-TS tested on the TT100K dataset. (**a**) YOLOv7; (**b**) YOLOv7-TS.

**Figure 22:**
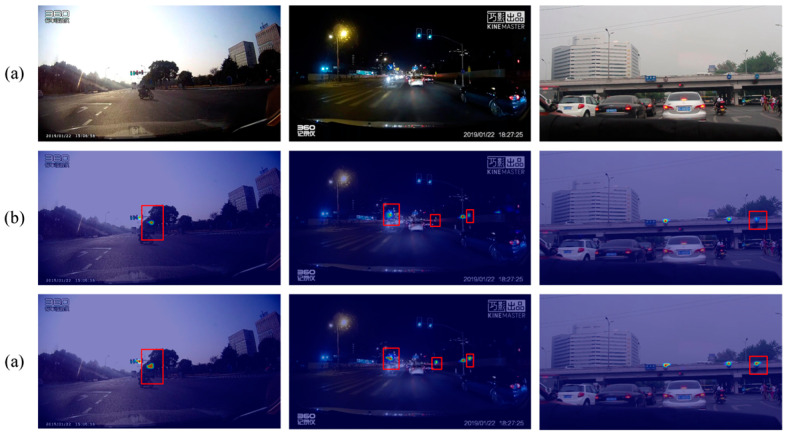
Heatmaps of YOLOv7 and YOLOv7-TS tested on the CCTSDB2021 dataset. (**a**) Original images; (**b**) YOLOv7. (**c**) YOLOv7-TS.

**Figure 23:**
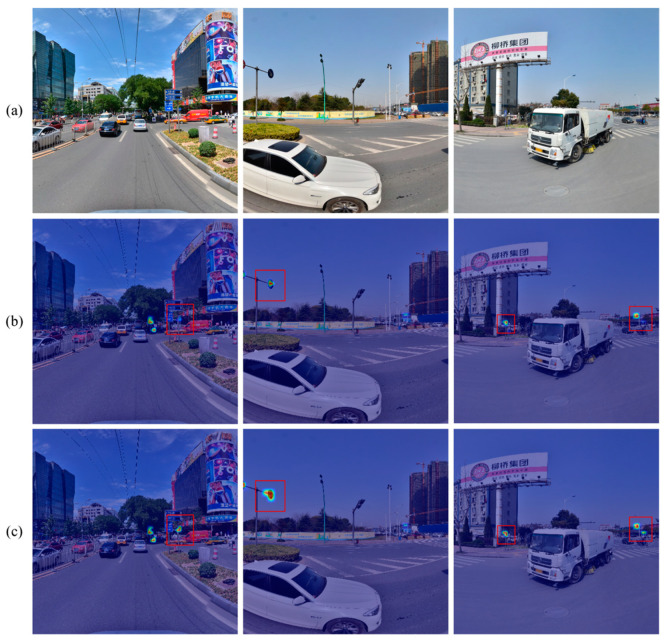
Heatmaps of YOLOv7 and YOLOv7-TS tested on the TT100K dataset. (**a**) Original images; (**b**) YOLOv7; (**c**) YOLOv7-TS.

For the sake of a clearer comparison of the detection results and regions of interest between the two models, the experimental results and heatmaps are visualized in Figure 20, Figure 21, Figure 22 and Figure 23. In Figure 20 and Figure 21, it is revealed that when traffic signs are very small, issues such as missing objects and low confidence in the detected objects are exhibited by YOLOv7. Conversely, higher confidence levels in object detection are consistently yielded by YOLOv7-TS, thus attesting to the successful mitigation of channel information loss and the enhancement of feature fusion. Furthermore, the objects that are overlooked by YOLOv7 are accurately detected by YOLOv7-TS, signifying its superior capability in perceiving small objects. In Figure 22 and Figure 23, the regions of interest and perceptual abilities of both models are depicted. The red boxes in the figures reflect that YOLOV7-TS has a stronger sensing ability than YOLOv7, enabling easier detection of nearby objects. With the incorporation of MIFNet, commendable perception capability for distant small objects is exhibited by YOLOv7-TS, whereas it remains a challenge for YOLOv7. In summary, the effectiveness of YOLOv7-TS in the realm of traffic sign detection is confirmed by the presented experimental results.

#### 4.4.2. Comparison Experiment between YOLOv7-TS and Other Popular Models

Comparative traffic sign detection experiments are also conducted between YOLOv7-TS and other common Two-Stage and One-Stage object detection algorithms using the CCTSDB2021 dataset, as shown in Table 8.

A significant advantage in terms of model performance and detection accuracy is exhibited by YOLOv7-TS compared with all other models. Specifically, Faster R-CNN, which utilizes a single feature layer without employing multi-scale feature fusion, achieves F1 and mAP values of 66.52% and 56.58%, respectively. Libra R-CNN, which is designed to pursue balance across three different levels during the training process, achieves F1 and mAP values of 69.93% and 61.35%, respectively. Dynamic R-CNN [52], which leverages dynamic label assignment strategies to adaptively adjust label assignment criteria, achieves F1 and mAP values of 69.83% and 60.01%, respectively. Although these Two-Stage algorithms show improved detection performance compared with R-CNN, they overlook feature enhancement, resulting in F1 and mAP values lower than those achieved by YOLOv7-TS. Furthermore, Two-Stage detection algorithms necessitate the generation of candidate regions prior to detection, leading to significantly slower detection speeds. In the case of One-Stage algorithms, the lowest performance is achieved by SSD, with F1 and mAP values of only 42.00% and 49.20%, respectively. This can be attributed to its direct use of multi-scale features for prediction by SSD while neglecting feature fusion and enhancement. The classic YOLOv3 algorithm achieves F1 and mAP values of 56.77% and 51.69%, respectively, and YOLOv4, achieves further improvements of 5.38% and 1.21%, respectively. Although FPN is employed for feature fusion in YOLOv3, it overlooks feature enhancement. Information with varying receptive fields is introduced through SPP in YOLOv4, but it does not match the feature enhancement achieved by DFEM. Class imbalance issues during the training of One-Stage algorithms are addressed by RetinaNet [53] through the introduction of focal loss, resulting in F1 and mAP values of 65.69% and 57.78%, respectively, surpassing those of YOLOv3 and YOLOv4. However, its detection speed struggles to meet real-time requirements. YOLOv5s achieves F1 and mAP values of 77.23% and 80.92%, respectively, while maintaining a high speed, while YOLOv5m sacrifices speed for higher detection accuracy. However, the issue of channel information loss during feature map extraction is not addressed by the YOLOv5 series, resulting in experimental results inferior to those of YOLOv7-TS. SC-YOLO [54], an improvement upon YOLOv5s, uses cross-stage attention networks and enhanced feature fusion, effectively improving small object detection capabilities and achieving F1 and mAP values of 84.50% and 84.30%, respectively. However, it still does not surpass the proposed YOLOv7-TS model. In summary, in testing on the CCTSDB2021 dataset, an F1 score of 84.90% and an mAP of 86.02% are achieved by YOLOv7-TS at 37.0 FPS, surpassing all other common algorithms. While it may not match the speed of YOLOv5s, YOLOv7-TS vastly outperforms it in terms of detection performance and accuracy.

## 5. Conclusions

In this study, we propose a traffic sign detection model named YOLOv7-TS, building upon YOLOv7 as its foundation. Firstly, the issue of feature channel information loss caused by the 1 × 1 convolution for feature map extraction is addressed through the introduction of FMEM. The feature maps extracted through FMEM simultaneously encompass high-level semantic information and low-level detailed information, actively promoting both object recognition and localization. Secondly, MIFNet is designed to enhance the feature fusion by promoting information interaction among all feature layers. Simultaneously, the rich detail information in the high-resolution feature map is combined to locate small objects and alleviate the problem of missing detection of small objects. Thirdly, DFEM enriches the highest-layer feature and improves the inference speed of the whole module. The highest-level feature containing global information and crucial information improves the detection accuracy of the model through feature fusion. In summary, YOLOv7-TS leverages the rich channel semantic information and spatial detail information in multi-scale features. Through feature enhancement and a more effective feature fusion network, the entire model benefits from these aspects. The analysis in the experimental section shows that YOLOv7-TS achieves higher F1 scores and mAP values compared with YOLOv7 and other common models. This fully demonstrates the excellent performance and highest detection accuracy of the model in traffic sign detection. The comparison of the results and heatmaps between YOLOv7-TS and YOLOv7 indicates that YOLOv7-TS alleviates the shortcomings of YOLOv7 in small object detection, showing a stronger ability to perceive small objects. However, in terms of FPS, our model lags behind YOLOv7 and YOLOv5s. This is attributed to the more complex architecture of YOLOv7-TS, requiring larger computational resources, resulting in a relatively slower inference speed. Our future work will focus on optimizing the model architecture, exploring the use of lightweight backbone networks and lightweight convolutions, while refining module details to reduce computational overhead and improve model speed.

This study was conducted based on traffic sign datasets. There are still many potential challenges and factors that should be considered when applying the model to real-world scenarios for traffic sign detection: Firstly, real-world environments exhibit diverse conditions. Changes in lighting due to different times of day and weather conditions may lead to variations in the appearance of traffic signs. Adverse weather, such as rain, snow, or haze, can blur or partially obstruct signs, making them more challenging to detect. Seasonal changes may alter road and environmental conditions, impacting sign visibility. Different road situations, such as wet, slippery, uneven, or dirty roads, may affect the clarity of traffic signs. Secondly, specific regions in the dataset may have distinct geographical and cultural characteristics compared with the target deployment area, posing challenges in detection due to regional variations. Thirdly, dynamic context adaptation is essential. Real-world scenarios often encompass dynamic contexts, such as urban versus rural environments or highways versus local roads. The model should dynamically adjust based on specific circumstances, considering factors such as traffic density, speed limits, and road infrastructure. Addressing these potential challenges requires the model to have higher robustness and stronger generalization capabilities when detecting in real-world scenarios. Several strategies can be employed: Firstly, data augmentation is crucial. Utilizing diverse data augmentation techniques, including random rotation, flipping, scaling, and brightness adjustments, helps simulate the diversity present in real-world scenarios. This aids in enhancing the model’s robustness to different environments and backgrounds. Secondly, training with multi-source data is essential. Integrating data from various regions and diverse scenarios ensures that the model encounters a broader range of contexts during the training phase. This contributes to improving the model’s generalization performance in different geographical and cultural environments. Thirdly, transfer learning is a valuable approach. Employing transfer learning methods allows the model to transfer knowledge learned in one region or scenario to another. This accelerates the adaptation process of the model to new environments. Fourth, it is beneficial to include an environment awareness module and real-time feedback. An environment awareness module is introduced into the model to capture and deal with environmental changes and adjust the detection strategy. The real-time feedback mechanism continuously monitors the performance of the model in real-world scenarios, enabling online adjustment based on actual feedback. Beyond adapting to diverse environments and backgrounds, deploying the model in real-world scenarios necessitates considerations of integration with existing systems, ethical concerns, and adherence to legal regulations. Seamless integration ensures effective deployment and collaboration with other components of traffic infrastructure. As artificial intelligence applications become more prevalent in real-world settings, compliance with ethical standards and legal regulations becomes paramount. Ensuring the model’s privacy compliance in public spaces is essential. In our future research, we will focus on addressing the potential challenges associated with deploying the model in real-world scenarios, continually enhancing the model’s robustness and generalization to deliver outstanding performance in various environments and contexts.

## Figures and Tables

**Table 1:** Results of the ablation study for FMEM.

Method	P (%)	R (%)	F1 (%)	mAP (%)
Methods on CCTSDB2021 dataset				
YOLOv7	92.11	70.45	79.84	82.39
YOLOv7 + FMEM	93.12	72.69	81.65	83.70
Methods on TT100K dataset				
YOLOv7	91.46	83.01	87.03	89.77
YOLOv7 + FMEM	91.78	84.17	87.81	90.71

**Table 2:** Results of the ablation study for MIFNet.

Method	P (%)	R (%)	F1 (%)	mAP (%)
Methods on CCTSDB2021 dataset				
YOLOv7	92.11	70.45	79.84	82.39
YOLOv7 + integration map	93.87	71.89	81.42	82.99
YOLOv7 + integration map + small object detection layer	90.34	78.21	83.84	83.91
YOLOv7 + MIFNet	92.23	78.36	84.73	84.02
Methods on TT100K dataset				
YOLOv7	91.46	83.01	87.03	89.77
YOLOv7 + integration map	92.89	83.78	88.10	90.12
YOLOv7 + integration map + small object detection layer	92.25	86.96	89.53	91.45
YOLOv7 + MIFNet	92.13	87.12	89.56	91.48

**Table 3:** Comparison of the parameters among several models.

Method	Params (M)
YOLOv7	36.9
YOLOv7 + integration map	35.1
YOLOv7 + integration map + small object detection layer	36.4
YOLOv7 + MIFNet	28.2

**Table 4:** Results of the ablation study for DFEM.

Method	P (%)	R (%)	F1 (%)	mAP (%)
Methods on CCTSDB2021 dataset				
YOLOv7	92.11	70.45	79.84	82.39
YOLOv7 + DFEM	94.24	71.23	81.14	83.71
Methods on TT100K dataset				
YOLOv7	91.46	83.01	87.03	89.77
YOLOv7 + DFEM	92.36	84.12	88.05	90.96

**Table 5:** Comparison of the parameters and inference time among several modules.

Method	Params (M)	Inference Time (s)
SPPCSPC	7.6	1.2542
DFEM (without pooling acceleration)	8.1	1.2787
DFEM	8.1	1.0588

**Table 6:** Results of the ablation study between the components in the proposed method.

Method	F1 (%)	mAP (%)
Methods on CCTSDB2021 dataset		
YOLOv7	79.84	82.39
YOLOv7 + FMEM + MIFNet	84.34	85.08
YOLOv7 + FMEM + DFEM	82.25	84.59
YOLOv7 + MIFNet + DFEM	83.96	84.83
YOLOv7+ FMEM + MIFNet + DFEM	84.90	86.02
Methods on TT100K dataset		
YOLOv7	87.03	89.77
YOLOv7 + FMEM + MIFNet	89.36	91.80
YOLOv7 + FMEM + DFEM	88.79	91.33
YOLOv7 + MIFNet + DFEM	89.50	91.95
YOLOv7+ FMEM + MIFNet + DFEM	89.92	92.45

**Table 7:** Results of the YOLOv7 and YOLOv7-TS.

Method	P (%)	R (%)	F1 (%)	mAP (%)	Params (M)	FPS
Methods on CCTSDB2021 dataset						
YOLOv7	92.11	70.45	79.84	82.39	36.9	42.3
YOLOv7-TS	92.69	78.31	84.90	86.02	34.7	37.0
Methods on TT100K dataset						
YOLOv7	91.46	83.01	87.03	89.77	36.9	43.1
YOLOv7-TS	92.36	87.60	89.92	92.45	34.7	37.3

**Table 8:** Results of the and YOLOv7-TS and different methods tested on the CCTSDB2021 dataset.

Method	F1 (%)	mAP (%)	FPS (%)
Faster R-CNN	66.52	56.58	4.9
Libra R-CNN	69.93	61.35	8.8
Dynamic R-CNN	69.83	60.01	9.0
SSD	42.00	49.20	22.3
YOLOv3	56.77	50.48	20.3
YOLOv4	62.15	51.69	16.6
RetinaNet	65.69	57.78	8.9
YOLOv5s	77.23	80.92	41.3
YOLOv5m	78.59	81.65	30.8
SC-YOLO	84.50	84.30	-
YOLOv7-TS	84.90	86.02	37.0

## Data Availability

The data presented in this study can be requested from the corresponding author, and these data are not currently available for public access.
